# Selective measurement of α smooth muscle actin: why β-actin can not be used as a housekeeping gene when tissue fibrosis occurs

**DOI:** 10.1186/s12867-017-0089-9

**Published:** 2017-04-27

**Authors:** Apor Veres-Székely, Domonkos Pap, Erna Sziksz, Eszter Jávorszky, Réka Rokonay, Rita Lippai, Kálmán Tory, Andrea Fekete, Tivadar Tulassay, Attila J. Szabó, Ádám Vannay

**Affiliations:** 1 MTA-SE Pediatrics and Nephrology Research Group, Budapest, Hungary; 2 1st Department of Pediatrics, Semmelweis University, Budapest, Hungary; 3 MTA-SE “Lendulet” Nephrogenetic Research Group, Budapest, Hungary; 4 MTA-SE “Lendulet” Diabetes Research Group, Budapest, Hungary

**Keywords:** Fibrosis, Primer design, Real-time PCR, Actin, α-SMA, ß-actin

## Abstract

**Background:**

Prevalence of fibroproliferative diseases, including chronic kidney disease is rapidly increasing and has become a major public health problem worldwide. Fibroproliferative diseases are characterized by increased expression of α smooth muscle actin (α-SMA) that belongs to the family of the six conserved actin isoforms showing high degree homology. The aim of the present study was to develop real-time PCRs that clearly discriminate α-SMA and ß-actin from other actin isoforms.

**Results:**

Real-time PCRs using self-designed mouse, human and rat specific α-SMA or ß-actin primer pairs resulted in the specific amplification of the artificial DNA templates corresponding to mouse, human or rat α-SMA or ß-actin, however ß-actin showed cross-reaction with the housekeeping γ-cyto-actin. We have shown that the use of improperly designed literary primer pairs significantly affects the results of PCRs measuring mRNA expression of α-SMA or ß-actin in the kidney of mice underwent UUO.

**Conclusion:**

We developed a set of carefully designed primer pairs and PCR conditions to selectively determine the expression of mouse, human or rat α-SMA and ß-actin isoforms. We demonstrated the importance of primer specificity in experiments where the results are normalized to the expression of ß-actin especially when fibrosis and thus increased expression of α-SMA is occur.

## Background

Incidence of chronic fibroproliferative diseases (FDs) is rapidly increasing and has become a major public health problem worldwide [1]. According to some estimates, about 45% of all deaths are attributed to FDs in the developed world [2].

The common hallmark of FDs is the activation of myofibroblasts (MFs), which produce excessive amount of extracellular matrix [2–4] leading to the destruction of original tissue architecture and gradual decline of organ function [5]. In response to activation, MFs express a high amount of α smooth muscle actin (α-SMA). Accordingly, measuring α-SMA expression is widely used to determine the presence and activity of MFs [6, 7].

α Smooth muscle actin belongs to the actin gene family consisting six different isoforms also including α-cardiac- and α-skeletal-actin, ß-actin, γ-cyto- and γ-smooth-actin. Beside α-SMA, ß-actin has a special importance also, as it is a widely used internal control in many molecular biological measurements. Although actin isoforms are encoded by different genes the similarity between them is significant. Indeed, the homology in the amino acid or nucleotide sequences of the different actin isoforms is over 90% making it a real challenge to selectively measure their expression [8]. Due to the increasing importance of FDs, the mRNA expression of α-SMA is frequently determined in thousands of experiments suggesting the importance of the issue. High accuracy, sensitivity and easy feasibility of real-time RT-PCR make it the most frequently used method to quantify gene expression in the field of basic and applied research as well [9–11]. Evidences suggest that real-time PCR enable the specific amplification of the target nucleotide sequence even if only one template molecule is present or if the difference is only one base from another nucleotide sequence [12]. In the present study we developed a SYBR Green stain based isoform-specific real-time PCR method to selectively measure the expression of mouse, human or rat α-SMA and ß-actin. Moreover, we investigated and unequivocally demonstrated the measurement inaccuracy caused by the use of non-specific α-SMA or ß-actin primer pairs in fibrotic kidney samples.

## Methods

### Design and alignment of α-SMA and ß-actin specific primers

**Fig. 1:**
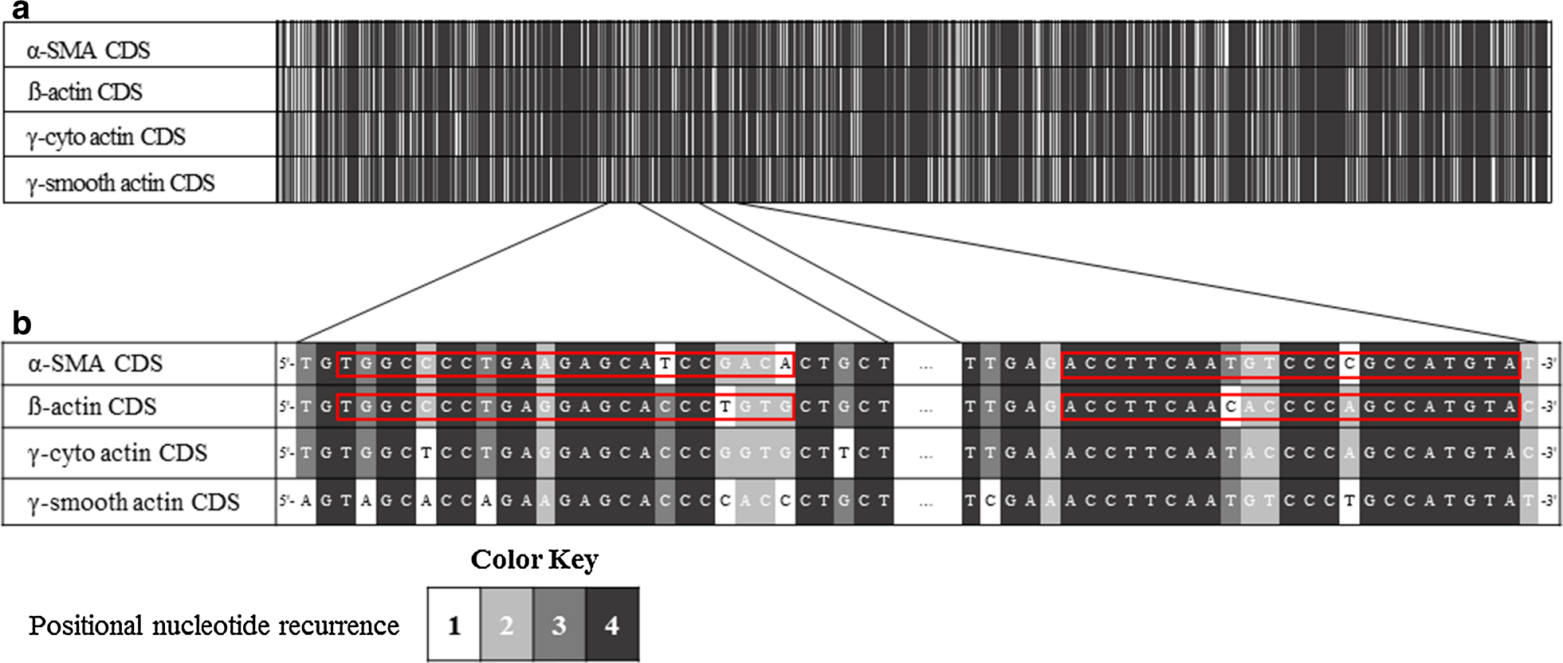
Heat map of nucleotide homology between CDSs of mouse α-SMA, ß-, γ-cyto- and γ-smooth-actin. Nucleotides were scaled from 1 to 4 individually according to their positional recurrence in the aligned CDSs of mouse α-SMA, mouse ß-actin, mouse γ-cyto-actin and mouse γ-smooth-actin (**a**). Priming sites of our mα-SMA_SD_ and mß-actin_SD_ primer pairs are enlarged and highlighted with *red boxes* (**b**)

The mRNA sequences of mouse α-SMA (NM_007392.3), human α-SMA (NM_001141945.2), rat α-SMA (NM_031004.2), mouse ß-actin (NM_007393.5), human ß-actin (NM_001101.3), rat ß-actin (NM_031144.3), mouse γ-cyto-actin (NM_009609.3) and mouse γ-smooth-actin (NM_009610.2) were collected from NCBI Reference Sequence Database [13]. Our self-designed mouse-, human- and rat α-SMA (mα-SMA_SD_, hα-SMA_SD_, rα-SMA_SD_) or ß-actin (mß-actin_SD_, hß-actin_SD_, rß-actin_SD_) specific primer pairs were designed by Primer3web software version 4.0.0 [14] considering the significant overlap between the mRNA sequences of different actin isoforms (Fig. 1; Table 1). Literary mouse α-SMA (mα-SMA_L1_ [15, 16], mα-SMA_L2_ [17, 18], mα-SMA_L3_ [19]) and ß-actin (mß-actin_L1_ [20, 21], mß-actin_L2_ [18], mß-actin_L3_ [22]) specific primer pairs were selected from papers published in different prestigious journals.

**Table 1:** Primer sequences and parameters

Name	Species	Primer pairs		Product length (bp)	T_a_ (°C)
mα-SMA_SD_	Mouse	F:	5′-CCCCTGAAGAGCATCGGACA-3′	105	60
		R:	5′-TGGCGGGGACATTGAAGGT-3′		
mß-actin_SD_	Mouse	F:	5′-CCCCTGAGGAGCACCGTGTG-3′	106	60
		R:	5′-ATGGCTGGGGTGTTGAAGGT-3′		
mα-SMA_L1_	Mouse	F:	5′-CACTATTGGCAACGAGCGC-3′	60	48
		R:	5′-CCAATGAAGGAAGGCTGGAA-3′		
mα-SMA_L2_	Mouse	F:	5′-GTCCCAGACATCAGGGAGTAA-3′	101	57
		R:	5′-TCGGATACTTCAGCGTCAGGA-3′		
mα-SMA_L3_	Mouse	F:	5′-GAGGCACCACTGAACCCTAA-3′	154	54
		R:	5′-CATCTCCAGAGTCCAGCACA-3′		
mß-actin_L1_	Mouse	F:	5′-GGCTGTATTCCCCTCCATCG-3′	154	56
		R:	5′-CCAGTTGGTAACAATGCCATGT-3′		
mß-actin_L2_	Mouse	F:	5′-TGTTACCAACTGGGACGACA-3′	165	55
		R:	5′-GGGGTGTTGAAGGTCTCAAA-3′		
mß-actin_L3_	Mouse	F:	5′-AGCCATGTACGTAGCCATCC-3′	228	57
		R:	5′-CTCTCAGCTGTGGTGGTGAA-3′		
RN18S	Mouse	F:	5′-AGCGGTCGGCGTCCCCCAACTTCT-3′	107	60
		R:	5′-GCGCGTGCAGCCCCGGACATCTA-3′		
hα-SMA_SD_	Human	F:	5′-ACTGAGCGTGGCTATTCCTCCGTT-3′	111	58
		R:	5′-GCAGTGGCCATCTCATTTTCA-3′		
hß-actin_SD_	Human	F:	5′-ACCGAGCGTGGCTACAGCTTCACC-3′	114	53
		R:	5′-AGCACCCGTGGCCATCTCTTTCTCG-3′		
rα-SMA_SD_	Rat	F:	5′-GAGCGTGGCTATTCCTTCGTG-3′	106	54
		R:	5′-CAGTGGCCATCTCATTTTCAAAGT-3′		
rß-actin_SD_	Rat	F:	5′-ACCGAGCATGGCTACAGCGTCACC-3′	106	54
		R:	5′-GTGGCCATCTCTTGCTCGGAGTCT-3′		

Nucleotide sequences of forward (F) and reverse (R) primers, product lengths and specific optimal annealing temperatures (T_a_) applied for the real-time PCR detection

### Artificial DNA templates

Artificial templates of mouse α-SMA (mα-SMA_T_), ß-actin (mß-actin_T_), γ-cyto-actin (mγ-cyto-actin_T_) and γ-smooth-actin (mγ-smooth-actin_T_) covering all of the annealing sections of the examined primers were synthetized as gBlocks Gene Fragments by Integrated DNA Technologies (Coralville, IA, USA). Human and rat α-SMA (hα-SMA_T_ and rα-SMA_T_) and ß-actin (hß-actin_T_ and rß-actin_T_) DNA templates were synthetized by PCR method using specific human or rat α-SMA and ß-actin primers. RT-PCR products were then separated by electrophoresis in 2% agarose gel. Thereafter, fractions with the required product length were extracted from the gel, purified by SureClean Plus purification kit (Bioline, Taunton, MA, USA) and resolved in RNase-free water.

### Unilateral ureteral obstruction (UUO) surgical protocol

The institutional committee on animal welfare approved all experiments (PEI/OO1/83-4/2013). Experiments were performed on 7–8 week old male C57BL/6J mice (Charles River Laboratories, Sulzfeld, Germany). Animals were housed in a temperature-controlled (22 ± 1 °C) room with alternating light and dark cycles and had free access to standard rodent chow and water. Mice were randomly divided into two groups (Control and UUO; n = 6/groups). After general anesthesia mice were placed on a thermo-controlled table to maintain rectal temperature at 37 ± 1 °C. After standard midline laparotomy, the bowel was gently displaced from the abdomen and covered with saline soaked sterile gauze. Then the left ureter of animals in the UUO group was isolated by blunt dissection and completely ligated using fine suture material. The bowel was then laid back and the muscle and skin were closed with 4–0 nylon sutures. Sham-operated control animals underwent identical surgical procedure without occlusion of the left ureter. Seven days after the initiation of UUO left kidneys were surgically removed, immediately snap-frozen and stored at −80 °C for further analysis.

### RNA isolation and cDNA synthesis

Total RNA was isolated from kidney samples of mice underwent UUO and sham-operated controls by Total RNA Mini Kit (Geneaid Biotech Ltd., New Taipei City, Taiwan) according to the instructions of the manufacturer. The concentration and quality of the isolated RNA was determined by DeNovix DS-11 spectrophotometer (DeNovix Inc., Wilmington, DE, USA). 500 ng of total RNA was reverse-transcribed using Maxima First Strand cDNA Synthesis Kit for RT-qPCR (Thermo Fisher Scientific, Waltham, MA, USA) to generate first-stranded cDNA.

### Real-time polymerase chain reaction

The expression of α-SMA and ß-actin was measured by real-time PCR on a Light Cycler 480 system (Roche Diagnostics, Mannheim, Germany). The reaction mix contained 10 pmol/μl of self-designed or literary forward and reverse PCR primers (Table 1; Integrated DNA Technologies, Coralville, Iowa, USA), 10 μl of Light Cycler 480 SYBR Green I Master enzyme mix (Roche Diagnostics, Mannheim, Germany) and 1 µl of the corresponding artificial DNA templates (0.1 nM) or cDNA. Nucleotide sequences of the applied primer pairs, their specific optimal annealing temperatures and product length are shown in Table 1. Results were analyzed by Light-Cycler 480 software version 1.5.0.39 (Roche Diagnostics, Mannheim, Germany). PCR products were separated by electrophoresis in 2% agarose gel (Bioline, London, UK) using 1X Tris–borate-EDTA buffer. Gels were stained with GelRed (Biotium, Hayward, Ca, USA) and were visualized and documented by VersaDoc 5000MP (Bio-Rad Laboratories, Hercules, CA, USA). Product lengths were determined using GeneRuler 100 bp DNA Ladder (Thermo Fisher Scientific, Waltham, MA, USA).

In each PCR the mRNA expression of α-SMA and ß-actin was determined by comparison with the expression of 18S ribosomal RNA (RN18S) as internal control from the same samples using the ∆∆Ct method. The data were normalized and presented as the ratio of their control values.

To determine the efficiency of real-time PCRs using mα-SMA_SD_ or mß-actin_SD_ primer pairs by a calibration curve, we applied a tenfold dilution series (from 10 nM to 1 fM) of mα-SMA_T_ or mß-actin_T_. The efficiencies were calculated by Light-Cycler 480 software version 1.5.0.39 (Roche Diagnostics, Mannheim, Germany).

### Sequencing of PCR products

The products of our α-SMA and ß-actin specific real-time PCRs amplifying cDNA samples derived from the kidneys of mice underwent UUO were purified by SureClean Plus purification kit (Bioline, Taunton, MA, USA) and sequenced using BrightDye Terminator Cycle Sequencing Kit (Nimagen, Nijmegen, The Netherlands) according to the instructions of the manufacturer. Sanger sequencing was performed on ABI 3500 sequencer (Thermo Fischer Scientific, Waltham, MA, USA) and chromatograms were analyzed by Unipro UGENE software version 1.16.1. (UniPro, Novosibrisk, Russia).

### Statistical analysis

The statistical evaluation and presentation of the normalized, relative mRNA expressions were performed by GraphPad Prism 6.01 software (GraphPad Software Inc., La Jolla, CA, USA). After testing normality with Kolmogorov–Smirnov test, unpaired *t* test or Mann–Whitney U test was used to determine the differences between the groups (Table 2). p ≤ 0.05 was considered as statistically significant. Values were expressed as mean + standard deviation (SD).

**Table 2:** Statistical analysis of mRNA expression of α-SMA and ß-actin in mice kidneys underwent UUO

Applied primer	Normality test		Comparison test (control vs. UUO)		
	Test	P value		Test	P value
		Control	UUO		
mα-SMA_SD_	Kolmogorov–Smirnov	0.2	0.2	Unpaired t test	<0.0001
mα-SMA_L1_	Kolmogorov–Smirnov	0.0115	0.2	Mann–whitney U test	0.0023
mα-SMA_L2_	Kolmogorov–Smirnov	0.2	0.2	Unpaired t test	<0.0001
mα-SMA_L3_	Kolmogorov–Smirnov	0.2	0.2	Unpaired t test	<0.0001
mß-actin_SD_	Kolmogorov–Smirnov	0.1283	0.2	Unpaired t test	0.4187
mß-actin_L1_	Kolmogorov–Smirnov	0.2	0.2	Unpaired t test	0.0022
mß-actin_L2_	Kolmogorov–Smirnov	0.2	0.2	Unpaired t test	0.0047
mß-actin_L3_	Kolmogorov–Smirnov	0.2	0.2	Unpaired t test	0.0021

The mRNA expression of α-SMA and ß-actin was determined by comparison with the expression of 18S ribosomal RNA (RN18S) as internal control from the same samples using the ∆∆Ct method

To determine the correlation between the relative mRNA expressions, Pearson correlation analysis was performed. Interpretation of Pearson correlation coefficient (r) [23] is summarized in Table 3. p ≤ 0.05 was considered as statistically significant.

**Table 3:** Interpretation of Pearson correlation coefficients

Size of correlation coefficient	Interpretation
0.9 ≤ r ≤ 1	Very high positive correlation
0.7 ≤ r ≤ 0.9	High positive correlation
0.5 ≤ r ≤ 0.7	Moderate positive correlation
0.3 ≤ r ≤ 0.5	Low positive correlation
0 ≤ r ≤ 0.3	Negligible correlation

## Results

### Template specificity of mα-SMA_SD_ and mß-actin_SD_ primer pairs

**Fig. 2:**
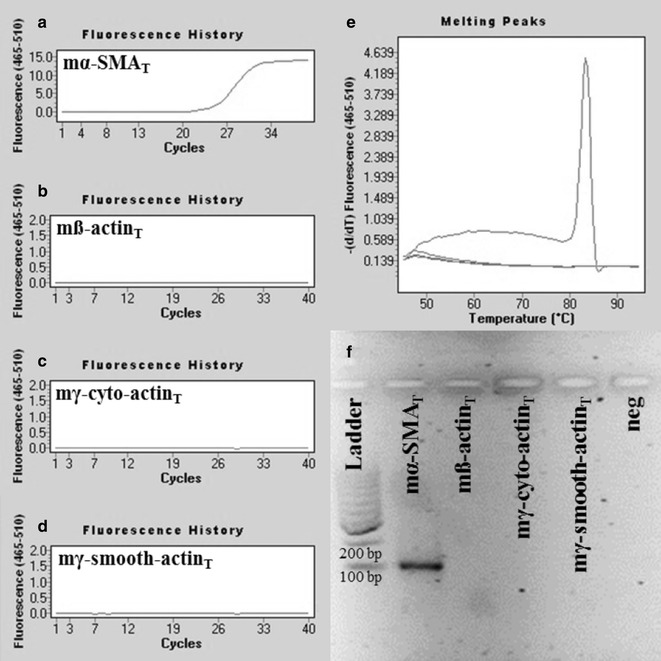
Representative plots of mouse α-SMA specific real-time PCRs. PCRs using mα-SMA_SD_ primer pair amplified mα-SMA_T_ artificial DNA template (Ct = 25.64) (**a**), but did not amplify the mß-actin_T_, mγ-cyto-actin_T_ and mγ-smooth-actin_T_ DNA fragments (**b**–**d**). Our mouse α-SMA specific PCR resulted in a product with single melting peak at 83.4 °C (**e**) and in one discrete band at 105 bp in agarose gel after electrophoresis (**f**)

**Fig. 3:**
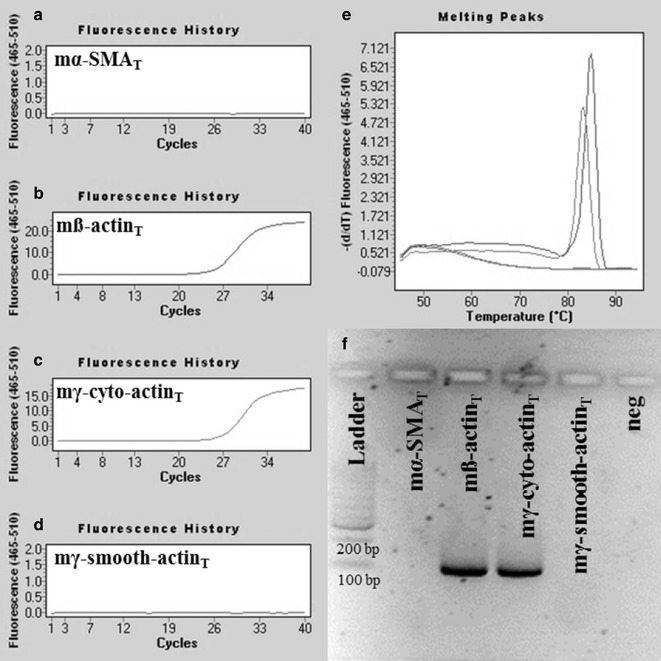
Representative plots of mouse ß-actin specific real-time PCRs. PCRs using mß-actin_SD_ primer pair amplified mß-actin_T_ (Ct = 15.76) and mγ-cyto-actin_T_ (Ct = 26.94) DNA (**b**, **c**), but did not amplify mα-SMA_T_ and mγ-smooth-actin_T_ DNA fragments (**a**, **d**). Our mouse ß-actin PCR resulted in products with melting peaks at 84.8 and 83.3 °C (**e**) and a discrete band at 106 bp in agarose gel after electrophoresis (**f**)

To investigate the specificity of mα-SMA_SD_ and mß-actin_SD_ primer pairs real-time PCR was performed where artificial DNA oligos corresponding to mouse α-SMA, ß-, γ-cyto- or γ-smooth-actin (mα-SMA_T_, mß-actin_T_, γ-cyto-actin_T_ and mγ-smooth-actin_T_) served as templates. According to our expectations, PCRs using our mα-SMA_SD_ primer pair amplified the mα-SMA_T_ DNA template and resulted in a product with single melting peak at 83.4 °C and a discrete band at 105 bp in agarose gel electrophoresis (Fig. 2a, e and f), but did not amplified mß-actin_T_, γ-cyto-actin_T_ and mγ-smooth-actin_T_ DNA templates (Fig. 2b–f). Similarly, PCRs using mß-actin_SD_ primer pair amplified the mß-actin_T_ and γ-cyto-actin_T_ DNA templates and resulted in the same products with melting peaks at 84.8 and 83.3 °C and bands at 106 bp (Fig. 3b, c, e and f), but did not amplify mα-SMA_T_ and mγ-smooth-actin_T_ DNA templates (Fig. 3a, d–f).

### Template specificity of mα-SMA_SD_ and mß-actin_SD_ primer pairs

**Fig. 4:**
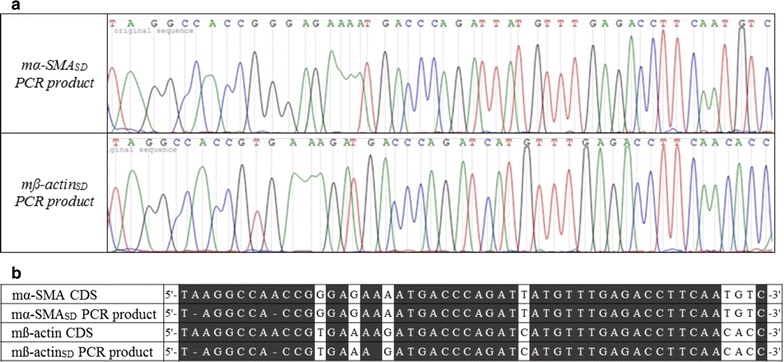
Sanger sequencing chromatograms of PCR products amplified by mα-SMA_SD_ and mß-actin_SD_ primer pairs. cDNA samples derived from kidneys of mice underwent unilateral ureteral obstruction were amplified using mα-SMA_SD_ or mß-actin_SD_ primer pair and products were sequenced (**a**). Nucleotide homology between the sequence of our PCR products and the known CDSs of mouse α-SMA or ß-actin was complete (**b**)

To investigate the specificity of our mα-SMA_SD_ and mß-actin_SD_ primer pairs, real-time PCRs were performed using cDNA templates generated from mice kidneys underwent ureteral obstruction. The subsequent Sanger sequencing of the PCR products demonstrated that the nucleotide sequences were identical with the reference nucleotide sequences (α-SMA: NM_007392.3; ß-actin: NM_007393.5) (Fig. 4).

### Amplification efficiency of real-time PCRs

**Fig. 5:**
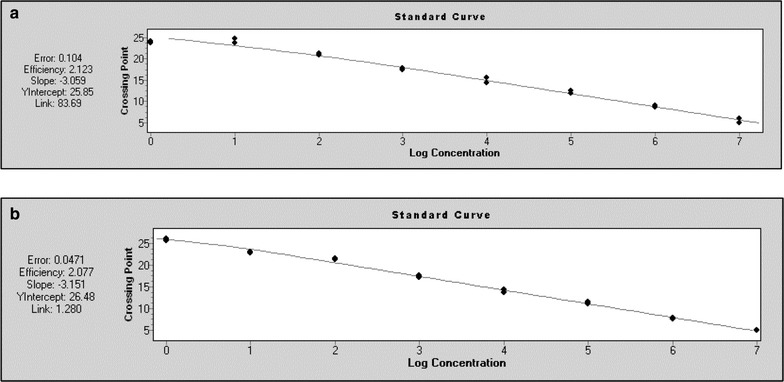
Amplification efficiency of real-time PCRs. Using mα-SMA_SD_ (**a**) or mß-actin_SD_ (**b**) primer pairs, tenfold dilution series of mα-SMA_T_ or mß-actin_T_ solutions served as template

Efficiencies derived from the slopes of calibration curves using mα-SMA_SD_ or mß-actin_SD_ were 2.123 or 2.077, respectively (Fig. 5).

### Specificity of literary primer pairs used to determine the expression of mouse α-SMA or ß-actin

**Fig. 6:**
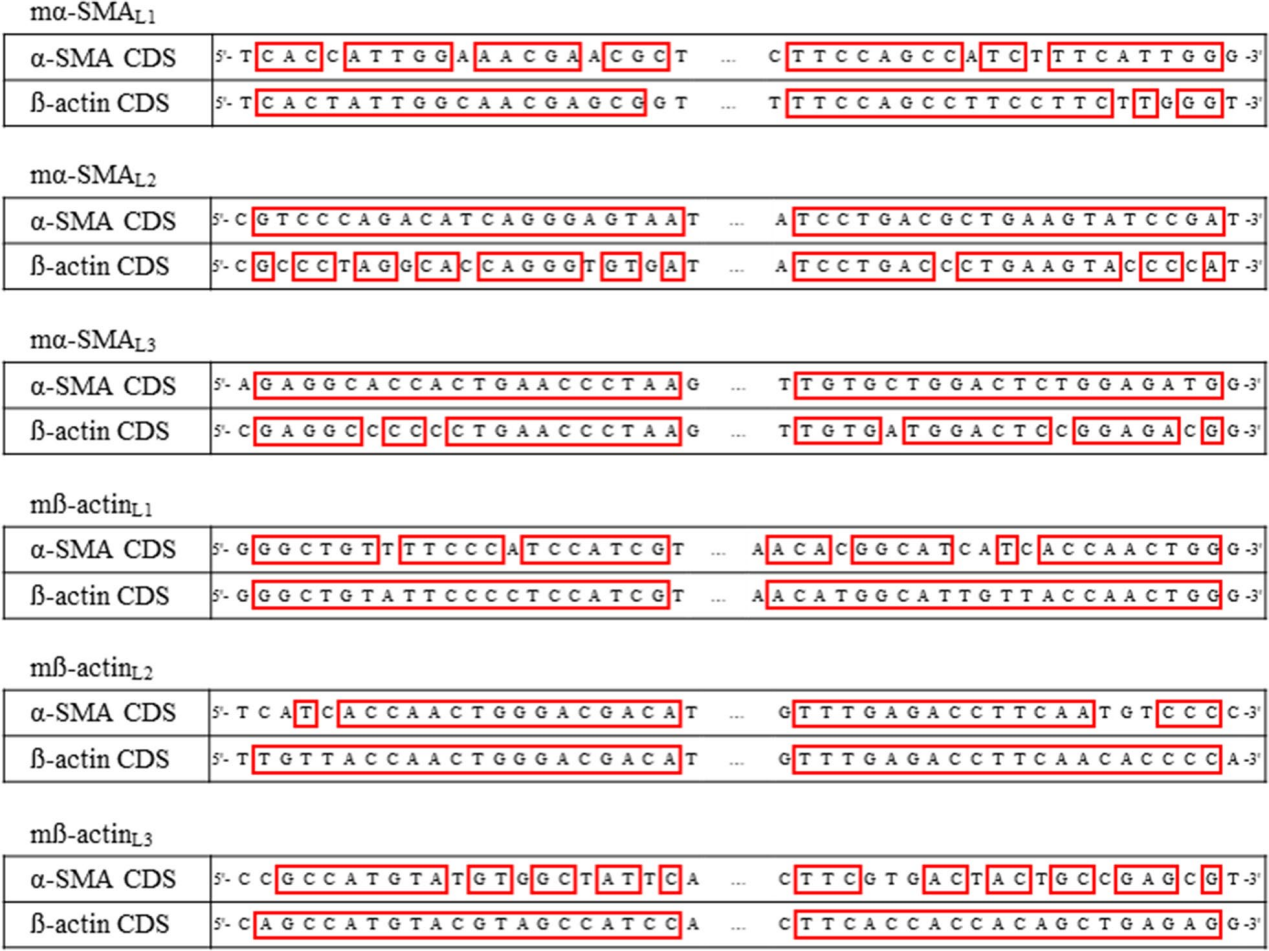
Priming sites of literary primer pairs aligned to the CDS of mouse α-SMA and ß-actin. Nucleotide sequence of CDSs corresponding to the examined literary primers are highlighted with *red boxes*

**Fig. 7:**
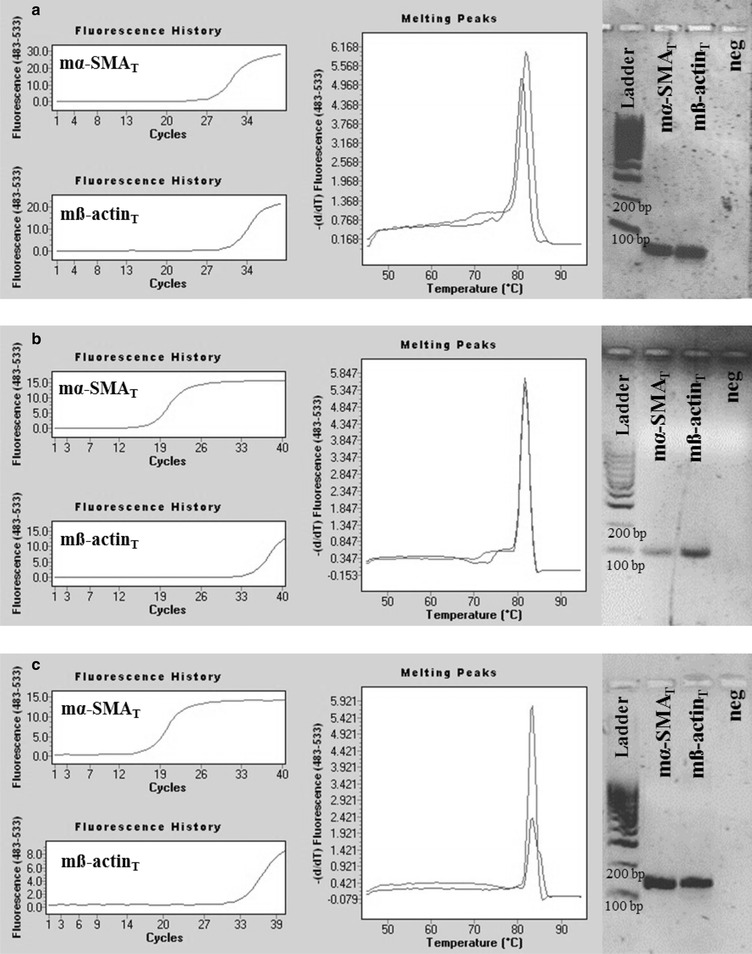
Representative plots of real-time PCRs using literary primer pairs specific for mouse α-SMA. Real-time PCRs using mα-SMA_L1_, mα-SMA_L2_ or mα-SMA_L3_ primer pairs and mα-SMA_T_ DNA templates resulted in products (Ct_L1_ = 28.09, Ct_L2_ = 17.19, Ct_L3_ = 16.79) with single melting peaks at 82, 81.9, or 83.3 °C and in discrete bands in the agarose gel at 60, 101 or 154 bp, respectively (**a**–**c**). Real-time PCRs using mα-SMA_L1_, mα-SMA_L2_ or mα-SMA_L3_ literary primer pairs amplified the non-specific mß-actin_T_ DNA templates also (Ct_L1_ = 31.09, Ct_L2_ = 34.62, Ct_L3_ = 32.96), resulted in melting peaks at 81, 81.9, or 83.3 °C and in discrete bands at 60, 101 or 154 bp, respectively (**a**–**c**)

**Fig. 8:**
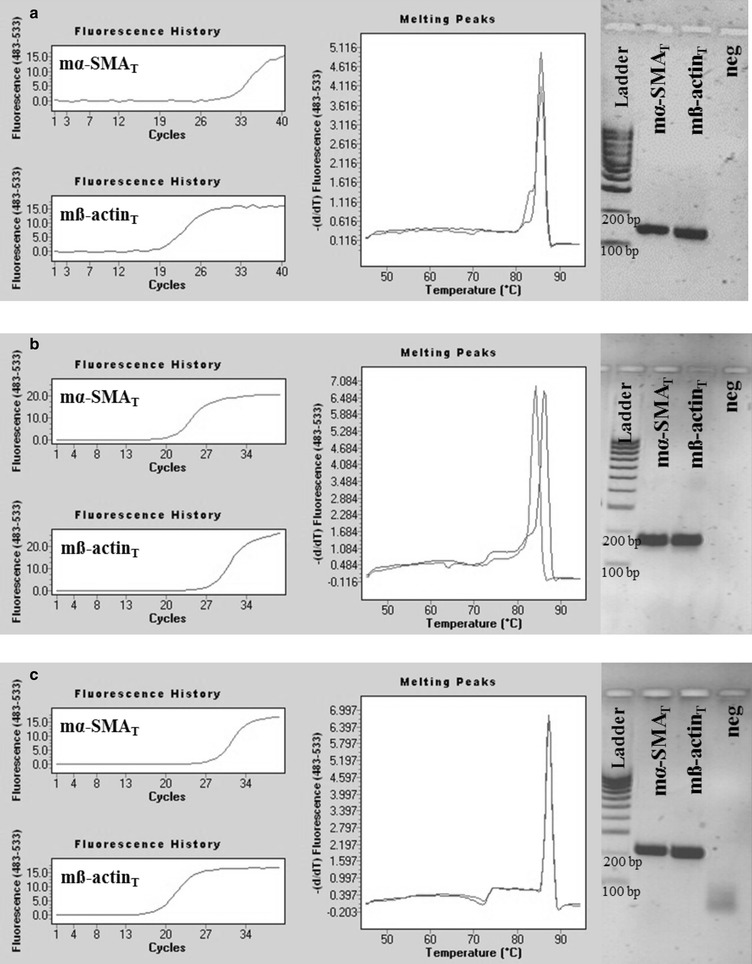
Representative plots of real-time PCRs using literary primer pairs specific for mouse ß-actin. Real-time PCRs using mß-actin_L1_, mß-actin_L2_ or mß-actin_L3_ primer pairs and mß-actin_T_ DNA templates resulted in products (Ct_L1_ = 19.73, Ct_L2_ = 27.72, Ct_L3_ = 18.65) with single melting peaks at 85.6, 86.3, or 87.4 °C and in discrete bands in the agarose gel at 60, 101 or 154 bp, respectively (**a**–**c**). Real-time PCRs using mß-actin_L1_, mß-actin_L2_ or mß-actin_L3_ literary primer pairs amplified the non-specific mα-SMA_T_ DNA templates also (Ct_L1_ = 31.29, Ct_L2_ = 21.08, Ct_L3_ = 28.43), resulted in melting peaks at 85.6, 84.2 or 87.4 °C and indiscrete bands at 60, 101 or 154 bp, respectively (**a**–**c**)

The nucleotide sequence of all literary primers show significant overlap with the coding sequence (CDS) both of α-SMA and of ß-actin (Fig. 6). To test the specificity of the literary primers (Table 1), real-time PCRs were performed using artificial DNA templates corresponding to mouse α-SMA and ß-actin (mα-SMA_T_, mß-actin_T_) as well. According to our expectations, all randomly selected literary mouse α-SMA (mα-SMA_L1_, mα-SMA_L2_ and mα-SMA_L3_) and ß-actin (mß-actin_L1_, mß-actin_L2_ and mß-actin_L3_) primer pairs amplified the corresponding specific DNA templates, but also showed a varying degree of cross-reactivity with the non-specific templates (Figs. 7, 8).

Real-time PCRs using mα-SMA_L1_, mα-SMA_L2_, or mα-SMA_L3_ primer pairs and mα-SMA_T_ DNA template resulted in products with single melting peaks at 82, 81.9, or 83.3 °C and one discrete band at 60, 101, or 154 bp in agarose gel electrophoresis (Fig. 7a–c). However, real-time PCRs using the same literary mouse α-SMA primer pairs unspecifically amplified mß-actin_T_ DNA templates also, resulted in the above listed melting peaks and electrophoretic bands, respectively (Fig. 7a–c).

Real-time PCRs using mß-actin_L1_, mß-actin_L2_, or mß-actin_L3_ primer pairs and mß-actin_T_ DNA template resulted in products with single melting peaks at 85.6, 86.3, or 87.4 °C and one discrete band at 60, 101, or 154 bp in agarose gel electrophoresis (Fig. 8a–c). However, real-time PCRs using the same literary mouse ß-actin primers unspecifically amplified mα-SMA_T_ DNA templates also, resulted in the above listed melting peaks and electrophoretic bands, respectively (Fig. 8a–c).

### mRNA expression of α-SMA and ß-actin in mice kidneys underwent UUO

**Fig. 9:**
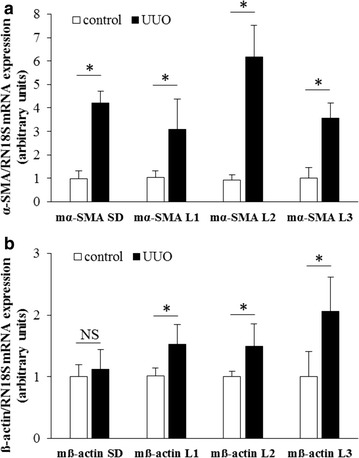
Comparison the results of real-time RT-PCRs investigating the mRNA expression of α-SMA or ß-actin using our or literary primer pairs. Depending on the applied primer pairs, the increase in mRNA expression of α-SMA varied from 3.1- to 6.2-fold in the kidneys of mice underwent UUO compared to the corresponding controls (**a**). While using our primer pair there was no difference in the mRNA expression of ß-actin, using literary primer pairs increased ß-actin mRNA expression was detected in the kidney of mice underwent UUO compared to the corresponding controls (**b**). Results were determined by comparison with RN18S and presented as mean + SD. *p < 0.05 UUO vs. control

To investigate the effect of the primer specificity on the experimental results, real-time RT-PCRs were performed on kidney samples of mice underwent UUO and sham-operated controls. Using our self-designed or one of the three literary α-SMA specific primer pairs, the increase of the α-SMA mRNA expression in the UUO group varied from 3.1- to 6.2-fold compared to the corresponding controls (Fig. 9a).

Similarly, marked differences were observed between the renal ß-actin mRNA expression values of the same kidney samples using the different literary or our self-designed ß-actin primers. Real-time RT-PCR using our mß-actin_SD_ primer pair showed no significant changes in the renal ß-Actin mRNA expression between the UUO and control groups (p = NS vs. control). On the contrary, significantly higher mRNA expression of ß-actin was observed in the UUO group in each real-time RT-PCR experiments using literary primer pairs (p < 0.05 vs. control) (Fig. 9b).

**Fig. 10:**
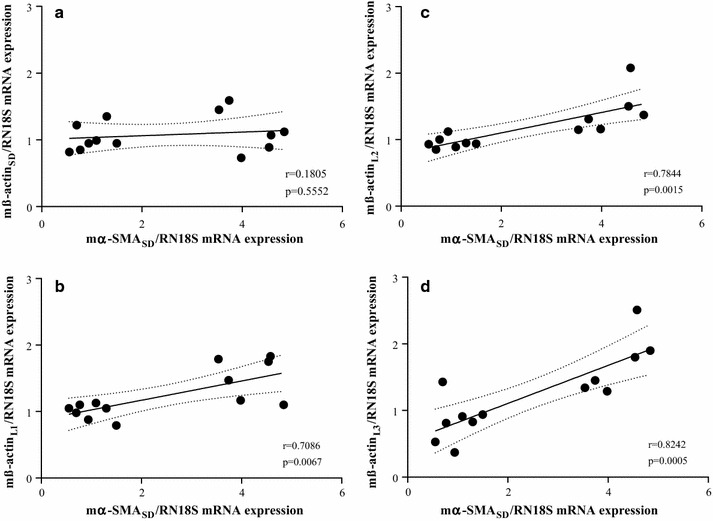
Correlation between relative α-SMA and ß-actin mRNA expression in mice kidneys. The relative ß-actin mRNA expressions measured by mß-actin_SD_ (**a**), mß-actin_L1_ (**b**), mß-actin_L2_ (**c**) or mß-actin_L3_ (**d**) primer pairs were correlated with the relative α-SMA expressions measured by mα-SMA_SD_ for each samples

Investigating the correlation between increased expression of α-SMA and ß-actin we found that there is only negligible, non-significant correlation between relative expression of α-SMA and ß-actin using our mß-actin_SD_ primers (r = 0.185, p = 0.5552, Fig. 10a). However, we found high positive, significant correlation in case of mß-actin_L1_ (r = 0.7086, p = 0.0067, Fig. 10b),

mß-Actin_l2_ (r = 0.7844, p = 0.0015, Fig. 10c) and mß-actin_L3_ (r = 0.8242, p = 0.0005, Fig. 10c) as well.

### Template specificity of hα-SMA_SD_, rα-SMA_SD_, hß-actin_SD_ and rß-actin_SD_ primer pairs

**Fig. 11:**
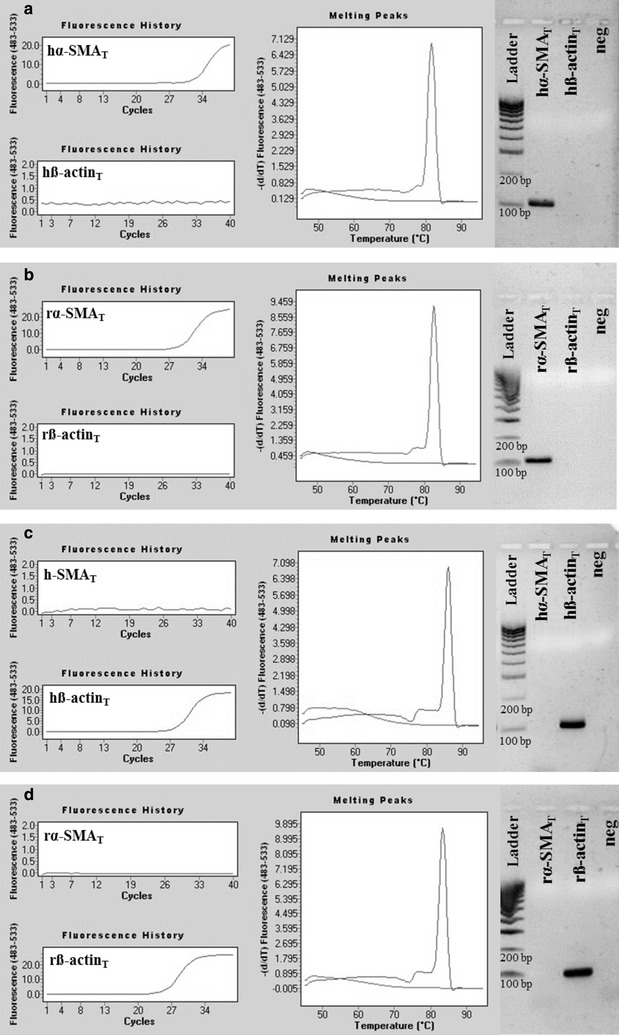
Representative plots of human and rat α-SMA and ß-actin specific real-time PCRs. PCRs using hα-SMA_SD_ or rα-SMA_SD_ specific primer pairs amplified the hα-SMA_T_ (Ct = 32) or rα-SMA_T_ (Ct = 28.21) DNA template (**a**, **b** respectively), but did not amplify the hß-actin_T_ or rß-actin_T_ template (**a**, **b** respectively). Our human or rat α-SMA specific PCRs resulted in products with melting peaks at 81.7 or 82.8 °C, and in discrete bands in agarose gel at 111 or 106 bp, respectively (**a**, **b**). Similarly, our hß-actin_SD_ or rß-actin_SD_ primer pair amplified the hß-actin_T_ (Ct = 27.42) or rß-actin_T_ (Ct = 25.58) DNA template (**c**, **d** respectively), but did not amplify the hα-SMA_T_ or rα-SMA_T_ template (**c**, **d** respectively). Our human or rat ß-actin specific PCRs resulted in products with melting peaks at 86 or 83.4 °C, and in discrete bands in agarose gel at 114 or 107 bp, respectively (**c**, **d**)

To investigate the specificity of our hα-SMA_SD_, rα-SMA_SD_, hß-actin_SD_ and rß-actin_SD_ primer pairs, real-time PCRs were performed using hα-SMA_T_, rα-SMA_T_, hß-actin_T_ or rß-actin_T_ DNA templates. According to our expectations, our hα-SMA_T_ and rα-SMA_T_ primer pairs amplified only the corresponding DNA template resulted in products with single melting peaks at 81.7 °C (Fig. 11a) or 82.8 °C (Fig. 11b), but did not amplified ß-actin DNA templates (Fig. 11a, b). Similarly, PCRs using our hß-actin_SD_ or rß-actin_SD_ primer pairs also amplified the corresponding DNA template only, resulted in products with single melting peaks at 86 °C (Fig. 11c) or 83.4 °C (Fig. 11d) but did not amplified the α-SMA DNA templates (Fig. 11c and d).

## Discussion

Family of actins comprises six different isoforms, among them α-SMA and β-actin have special importance. Recognizing the medical importance of FDs, the number of studies measuring the expression of α-SMA, the biomarker of the MFs, is increasing. Indeed, currently there in no study investigating the pathomechanism of organ fibrosis or aiming the development of new chemical compounds to inhibit fibrosis without measuring the expression of α-SMA. The other isoform of major importance is ß-actin which is one of the most frequently used housekeeping genes in thousands of studies every year. Although different actin isoforms are encoded by different genes, the homology in their amino acid or nucleotide sequences is over 90% making it challenging to determine the expression of the given isoform without cross-reactions with the others (Fig. 1) [8].

In the present study, we developed an isoform-specific real-time PCR method to selectively measure the mRNA expression of mouse, human and rat α-SMA and ß-actin as well. Moreover, we demonstrated the significance of the inaccuracy caused by the use of non-specific α-SMA or ß-actin primer pairs in the most frequently used mice model of renal fibrosis.

During PCRs DNA polymerase enzymes start the synthesis of the new DNA strand from the 3′ end of the annealed primers [24]. Mismatches in the 3′ end of the primers significantly determine the proper primer-annealing, and as consequence the specificity of the PCRs [25, 26]. Therefore, we located our different actin primers to nucleotide sequences with the greatest possible difference from other actin isoforms, paying special attention to the 3′ end of our primers to maximize the chance of specific priming (Fig. 1).

In the first set of experiments, target specificity of our mouse α-SMA and ß-actin specific primer pairs were tested (mα-SMA_SD_ and ß-actin_SD_, respectively) using chemically synthetized mouse α-SMA, ß-, γ-cyto- and γ-smooth-actin gene fragments as templates. Real-time PCRs using specific template of a certain primer pair resulted in products with one separate melting curve maximum, and likewise, in one discrete band with the expected product length during separation by gel electrophoresis. Applying our mouse α-SMA specific primer pairs in real-time PCRs containing the non-specific ß-, γ-cyto- or γ-smooth-actin DNA templates (Fig. 2), or our mouse ß-actin specific primer pair in real-time PCRs containing α-SMA, or γ-smooth actin specific DNA templates, PCR products were not observed (Fig. 3). However, due to the remarkable (nearly 90%) sequence homology between mouse ß- and γ-cyto-actin, we could not eliminate the cross-reaction between our mouse ß-actin primer pair and artificial DNA template corresponding to γ-cyto-actin. Fortunately, the biological significance of this cross-reaction is small, since both ß- and γ-cyto-actin are housekeeping genes, and the measurement of these as internal controls can be easily replaced by measuring another one [1]. Taken together, these observations demonstrate that our actin isoform specific primer pairs are suitable for the measurement of the mRNA expression of mouse α-SMA and ß-actin, respectively.

In the next step of PCR-validation process, we determined amplification efficiency of our real-time PCRs, using mα-SMA_SD_ or mß-actin_SD_ primer pairs. We found that amplification factors derived from the slopes of calibration curves were in the acceptable range from 1.8 to 2.2 (Fig. 5) [27, 28].

In the second set of experiments, the specificity of our mouse α-SMA and ß-actin primer pairs were tested on kidney samples of mice underwent UUO, which is a well-characterized experimental model of renal fibrosis. It is already well known that after the onset of UUO the number of α-SMA expressing MFs is increasing in the kidney in parallel with the development of fibrosis. The simultaneous strong expression of α-SMA and ß-actin makes the fibrotic kidney an excellent biological sample to test the possible cross-reaction of our primers and the different actin isoforms. The products of the mouse α-SMA and ß-actin specific real-time RT-PCR were sequenced, and the resulting nucleotide sequences were compared to the CDS of the corresponding actin isoforms (Fig. 4). The nucleotide sequences of these PCR products were identical to the reference CDS of the amplified genes, and did not show any overlap with the other actin isoform confirming the specificity of our primer pairs. Taken together, all these data suggest that our mouse α-SMA and ß-actin primers are clearly applicable to specifically measure the mRNA expression of mouse α-SMA and ß-actin.

In the third set of experiments, investigating the biological relevance of the non-specific primer binding, the template specificity of three mouse α-SMA and three ß-actin literary primer pairs were tested. As it was expected, based on the high degree of homology of these primers and the corresponding CDSs (Fig. 6), we found that all primer pairs amplified both mouse α-SMA and ß-actin specific artificial DNA templates as well (Figs. 7, 8). To investigate the relevance of non-specific primer binding on the experimental results, real-time RT-PCRs were performed on kidney samples of mice underwent UUO and sham-operated controls using the different set of primer pairs. Carried out the measurement with our primer pair, 4.2 fold relative increase was observed in the mRNA expression of α-SMA in the kidney of mice underwent UUO compared to the controls. In the cases of literary primer pairs, the fold change values of α-SMA mRNA expression varied from 3.0 to 6.2 in the same kidney samples (Fig. 9a), suggesting that the cross-reactions of these primers with the different actin isoforms may substantially alter the experimental results. Similarly, while using our carefully designed primer pair, the mRNA expression of ß-actin was equal in the two groups, applying the literary primer pairs it showed significant increase in the kidney of mice underwent UUO compared to controls (Fig. 9b). These results suggest that cross-reaction between ß-actin primer pairs and different actin isoforms, such as α-SMA in the fibrotic kidney, may lead to the virtually increased expression of ß-actin, the frequently measured housekeeping gene, in certain experimental conditions [29]. Our observation was confirmed by the high positive, significant correlation between the α-SMA expression measured by our primer pair (m-αSMA_SD_) and ß-actin expression measured by not properly designed literary primer pairs (Fig. 10). As a consequence of the inaccurate determination of the housekeeping gene expression it may result in false experimental outcomes and conclusions.

Given the importance of the issue, we also developed human and rat α-SMA and ß-actin specific primer pairs. The specificity of these primer pairs were tested in real-time PCRs using artificial DNA templates corresponding to human or rat α-SMA and ß-actin similarly to that described above. Results of these experiments suggest that our primer pairs can specifically bind to their target DNA allowing the specific measurement of the mRNA expression of human and rat α-SMA and ß-actin as well (Fig. 11).

## Conclusions

In summary, as the number of studies investigating the behavior of the MFs—the main effector cells of fibrosis—is increasing, so get more important the precise determination of α-SMA expression. In the present study we developed a set of carefully designed mouse, human and rat α-SMA specific primer pairs to determine the expression of α-SMA without cross reactions with other highly homologue actin isoforms. Our study also give an experimental explanation, how the cross reaction between different actin isoforms can influence the measurements concerning the expression of housekeeping gene ß-actin, underlining the importance of proper primer design.

## Abbreviations

α-SMA: α smooth muscle actin
bp: base pair
CDS: coding sequence
Ct: threshold cycle
FD: fibroproliferative disease
h: human
L: literary
m: mouse
MF: myofibroblast
r: rat
RN18S: 18s ribosomal RNA
SD: self-designed
T: artificial DNA template
UUO: unilateral ureteral obstruction
